# Deeper Insight in Beehives: Metagenomes of Royal Jelly, Pollen, and Honey from Lavender, Chestnut, and Fir Honeydew and Epiphytic and Endophytic Microbiota of Lavender and Rose Flowers

**DOI:** 10.1128/genomeA.00425-17

**Published:** 2017-06-01

**Authors:** Julien Crovadore, François Gérard, Romain Chablais, Bastien Cochard, Karl Kristian Bergman Jensen, François Lefort

**Affiliations:** a Plants and Pathogens Group, Research Institute Land Nature and Environment, hepia, HES-SO University of Applied Sciences and Arts Western Switzerland, Jussy, Geneva, Switzerland; b Boswellia SARL, Châteauneuf de Galaure, France; c New Nordic HealthBrands AB, Malmö, Sweden

## Abstract

Microbiota of beehive products are very little known. We report here for the first time six metagenomes of royal jelly, pollen, and different types of honey from wild and cultivated lavender, chestnut, and fir honeydew. Four metagenomes of epiphytic and endophytic microbiota of lavender and rose flowers are also reported.

## GENOME ANNOUNCEMENT

While the health benefits of beehive products are widely acknowledged, their associated microbiota remain very little known. Metagenomic studies are mainly focused on the microbiota of bee gut as a model for studying insect gut and for bee health issues (1, 2). A honey metagenome produced by pyrosequencing has been reported once (3), but other beehive products had not been investigated until now. Metagenomic shotgun sequencing allowed for describing the microbiota of beehive-related products such as honey (from wild and cultivated lavender, chestnut, and fir honeydew), royal jelly, and pollen. We also described the epiphytic and endophytic microbiota of rose and lavender flowers. 

All samples were taken from the mountains of the National Park of Mercantour, located north of La Bollène Vésubie (France), except one from a culture of *Lavendula latifolia* ×* officinalis* from the plateau of Valensole (located south of the National Park of Mercantour) and one from fir (*Abies alba*) honeydew honey from the Vosges mountains (Allarmont, France). Flowers of wild lavender (*Lavandula angustifolia*) were sampled in the mountains of La Bollène Vésubie, while flowers of *Rosa* × *damascena* were from La Pallud-sur-Verdon (France). Total DNA extractions from the microbiota of the honey samples were performed according to an adapted protocol of DNA extraction from honey (4), starting from 50 g of honey. Resulting pellets were extracted according to a CTAB-based protocol (5). DNA of epiphytic microbiota from lavender and rose flowers were extracted by incubating flowers in PBS (1×) 0.15% Tween 20 with shaking (400 rpm) for 10 min, followed by a 5-min sonication step, shaking for 10 min (400 rpm) prior to prefiltration, and filtration on a 0.22-µm sterile filter (Millipore, Germany). DNA was finally extracted from the filters using the CTAB-based protocol (5). DNA from the endophytic microbiota was extracted from 200 mg of fresh material using the same protocol (5), as was DNA from fresh pollen and royal jelly. All DNA isolations included an RNA digestion step with RNase A/T1 (Ambion RNase cocktail) and were re-suspended in RNase-DNase-free sterile water. For each sample, 0.1 to 1 µg of DNA was sheared in an AFA microTUBE (Covaris, USA) in an S2 ultrasonicator (Covaris) to achieve an average fragment size of 350 bp. Libraries were created using the TruSeq DNA PCR-free and TruSeq Nano DNA library preparation kits (Illumina, USA), and the insert size was checked in a Fragment analyzer (Advanced Analytical Technologies, Inc., USA). Whole-metagenome shotgun sequencing was carried out within three high-output (300 cycles) Illumina MiniSeq runs with a 2 × 151-bp paired-end read length. Reads were extracted from BaseSpace (Illumina) with an automatic trimming of the adaptor plus removal of Ns. The sequencing yield ranged from 0.13 to 4.37 Gb per sample. Quality control was performed with FastQC (http://www.bioinformatics.babraham.ac.uk/projects/fastqc). Bioinformatics analysis and operational taxonomic unit identification were performed with the metagenome classifiers “one Codex” (6), Kaiju (7), and the MG-RAST pipeline (8). *Lactobacillus kunkeei* was found dominant in honey, as observed by Asama (3), and in other royal jelly and pollen samples, too.

### Accession number(s).

Metagenome raw sequencing data have been made publicly available through the Sequence Read Archive (SRA) (9) of the National Center for Biotechnology Information under the SRA accession numbers given in Table 1. They have also been deposited at the MG-RAST database (accessible at http://metagenomics.anl.gov).

**TABLE 1:** Nucleotide sequence accession numbers

Sample name	Sequence Read Archive accession no.
*Rosa* × *damascena* epiphytic microbiota	SRR5170319
*Rosa × damascena* endophytic microbiota	SRR5172675
*Lavandula angustifolia* epiphytic microbiota	SRR5172868
*Lavandula angustifolia* endophytic microbiota	SRR5172873
*Castanea sativa* honey	SRR5172884
Fresh pollen from beehive	SRR5172883
Royal jelly	SRR5172922
*Lavandula angustifolia* honey	SRR5188317
*Lavendula latifolia* × *officinalis* honey	SRR5188336
Honeydew honey from *Abies alba*	SRR5208578
